# A case of ectopic pregnancy with a negative urine pregnancy test diagnosed preoperatively as ovarian hemorrhage

**DOI:** 10.20407/fmj.2025-033

**Published:** 2026-05-14

**Authors:** Ryoma Aoki, Akira Yasue, Yutaka Torii, Ryuichiro Aoki, Kazuhiko Tsukada, Haruki Nishizawa

**Affiliations:** 1 Department of Obstetrics and Gynecology, Fujita Health University, School of Medicine, Toyoake, Aichi, Japan; 2 Department of Gynecology, Fujita Health University Okazaki Medical Center, Okazaki, Aichi, Japan

**Keywords:** Negative urine pregnancy test, Ectopic pregnancy, Ovarian hemorrhage

## Abstract

**Introduction:**

Ectopic pregnancy is a typical obstetric emergency, and serum and urine human chorionic gonadotropin (hCG) measurements are important for its diagnosis. However, qualitative urine hCG tests sometimes give false-negative results. We herein report a case in which pregnancy was ruled out due to a negative qualitative urine hCG test, and surgery was performed based on a preoperative diagnosis of ovarian hemorrhage. However, the pregnancy was ultimately diagnosed as ectopic.

**Case Presentation:**

The patient was a 19-year-old woman, gravida 0, para 0. She presented to a local clinic for lower abdominal pain that occurred 28 days after her last menstrual period. She was then transferred to our hospital following confirmation of peritoneal irritation signs and abdominal computed tomography (CT) findings revealing ascites. She presented with severe tenderness and rebound tenderness around the umbilicus, and transvaginal ultrasonography revealed an 83-mm hematoma in the Douglas pouch. Since her qualitative urine hCG test was negative and contrast-enhanced CT scans revealed contrast leakage around the left adnexa, a diagnosis of left ovarian hemorrhage was made. Subsequently, emergency laparoscopic surgery was performed. However, the intraoperative finding of left fallopian tube enlargement led to suspicion of an ectopic pregnancy, prompting a change in the surgical procedure to left salpingectomy. Preoperative serum hCG level was elevated at 314 mIU/mL, and pathological examination confirmed chorionic villi, confirming a left fallopian tubal pregnancy.

**Conclusion:**

We experienced a case of ectopic pregnancy with a negative urine pregnancy test. In women of reproductive age presenting with an acute abdomen, the possibility of ectopic pregnancy should always be considered, even if the qualitative urine hCG test is negative, and careful informed consent is required.

## Introduction

Ectopic pregnancy is a typical obstetric emergency and is commonly encountered in clinical practice. Its incidence is estimated to be approximately 1%–2% of all pregnancies. Moreover, it is one of the most important differential diagnoses for acute abdomen in women of reproductive age. Approximately 96% of ectopic pregnancies occur in the fallopian tubes, with others occurring in sites such as the ovaries, abdominal cavity, and cervix.^1^ The clinical manifestations of ectopic pregnancy typically include amenorrhea, lower abdominal pain, and vaginal bleeding, with only approximately 50% of cases presenting with all three manifestations. The highly diverse pathophysiology of ectopic pregnancy can make diagnosis challenging. If tubal abortion or tubal rupture occurs, it presents as an acute abdomen, requiring prompt diagnosis and appropriate treatment.

On the other hand, ovarian hemorrhage, a condition that must be differentiated from ectopic pregnancy, is characterized by intraovarian or extraovarian hemorrhage during the ovulatory and luteal phases or during pregnancy. Similar to ectopic pregnancy, intraperitoneal hemorrhage can cause acute lower abdominal pain and anemia.

When differentially diagnosing ectopic pregnancy and ovarian hemorrhage, measurement of serum or urine human chorionic gonadotropin (hCG) levels is important to confirm the presence of pregnancy. Serum hCG tests, in particular, have high sensitivity and specificity. Meanwhile, qualitative urine hCG tests can occasionally yield false-negative results. Because of their lower sensitivity than serum hCG tests, cases have been reported in which the serum hCG test was positive but the qualitative urine hCG test was negative.^2^ In such cases, pregnancy is likely to be ruled out, making it difficult to differentiate between the two conditions.^3^

Herein, we report a case in which pregnancy was ruled out due to a negative preoperative qualitative urine hCG test, prompting emergency surgery based on the diagnosis of ovarian hemorrhage. However, intraoperative findings revealed an ectopic pregnancy. This case highlights the diagnostic challenges of ectopic pregnancy in hCG-negative cases and underscores the importance of detailed analysis of medical history and intraoperative findings.

## Case Presentation

The patient was a 19-year-old woman, gravida 0, para 0. She had no notable medical or family history. Her menstrual cycle was a regular 28-day cycle. On the 28th day after her last menstrual period, she complained of lower abdominal pain and visited a local gynecology clinic, where no abnormality was noted. However, due to recurrent abdominal pain during the night of that day, she visited a local gastroenterology clinic the next day. Peritoneal irritation signs were confirmed, and abdominal computed tomography (CT) imaging revealed signs of ascites, leading to her emergency transportation to our hospital.

**Figure 1:**
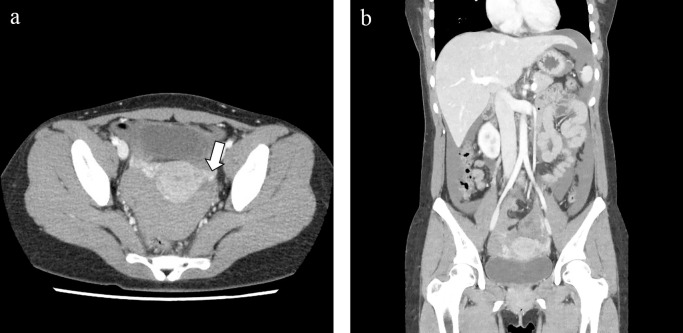
Abdominal contrast-enhanced CT scan: a) Findings suggestive of contrast extravasation (⇨) were observed around the left adnexa. b) A large volume of ascites was observed extending around the liver and spleen.

**Figure 2:**
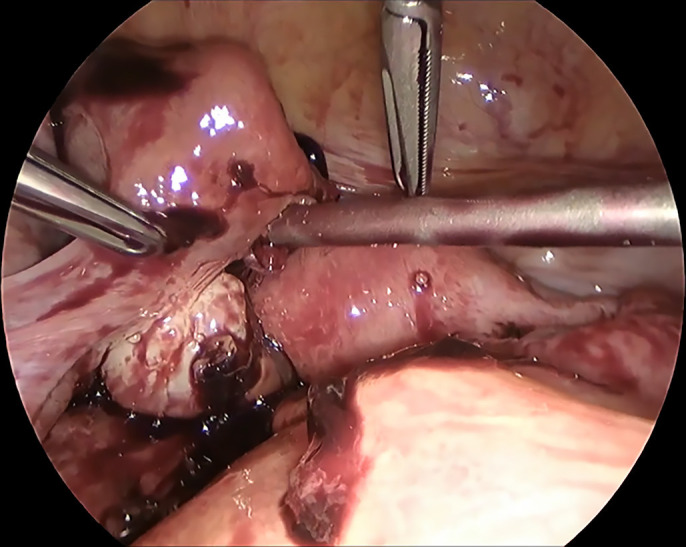
Surgical findings At the beginning of surgery, a large hematoma was observed in the Douglas pouch. Left fallopian tube: The left fallopian tube was markedly enlarged.

**Figure 3:**
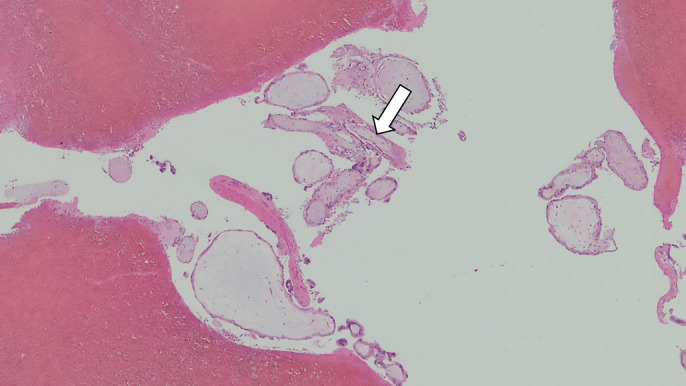
Histopathological findings A small amount of chorionic villi (⇨) were observed in the enlarged left fallopian tube, but no capillary development or fetal component were noted.

On examination, the patient was alert, with a blood pressure of 109/60 mmHg and a pulse rate of 111 beats/minute. Physical examination revealed severe tenderness and rebound tenderness around the umbilical region. Transvaginal ultrasonography revealed no evidence of endometrial thickening or gestational sac, while an 83-mm hematoma-like mass was observed in the Douglas pouch. Blood tests revealed mild inflammation and anemia with a white blood cell count of 17,300/μL, CRP at 1.11 mg/dL, and Hb of 10.2 g/dL, and coagulation tests revealed a mildly prolonged PT-INR of 1.18. Following a negative qualitative urine hCG test to rule out pregnancy, a contrast-enhanced CT scan was performed, which revealed ascites extending into the upper abdomen and findings suggestive of contrast extravasation around the left adnexa. Meanwhile, the right ovary was normal (Figure 1). Based on the negative qualitative urine hCG test, the possibility of ectopic pregnancy was ruled out at that time. A diagnosis of left ovarian hemorrhage was made, prompting emergency laparoscopic surgery. Intraoperative findings revealed a hematoma in the Douglas pouch with intraperitoneal hemorrhage of approximately 500 mL (Figure 2). There was no evidence of bilateral ovarian enlargement or hemorrhage, ruling out the possibility of ovarian hemorrhage. However, the left fallopian tube was enlarged, and the macroscopic findings were consistent with ectopic pregnancy. Therefore, despite the negative qualitative urine hCG test, the possibility of ectopic pregnancy was considered. Intraoperatively, informed consent was obtained from the patient’s family regarding the diagnosis and a change in surgical procedure. Subsequently, laparoscopic left salpingectomy was performed. No macroscopically apparent villous component was detected in the resected specimen. Postoperatively, the serum hCG level in the preoperative sample was confirmed to be 314 mIU/mL, and histopathological examination confirmed chorionic villi, ultimately confirming left fallopian tubal pregnancy (Figure 3). The patient’s postoperative course was uneventful, and she was discharged. At an outpatient visit 1 month after surgery, her serum hCG level was confirmed to be below the measurement sensitivity, and follow-up was discontinued.

## Discussion

An ectopic pregnancy is a pregnancy in which a fertilized egg implants outside the uterine cavity, causing intraperitoneal hemorrhage that can lead to shock and death. In recent years, the incidence of ectopic pregnancy has increased to approximately 2% due to the aging of the maternal population and an increase in pregnancies with a history of cesarean section and those resulting from assisted reproductive technologies. The most common site of implantation is the fallopian tube, accounting for approximately 96% of all ectopic pregnancies. Other sites include the abdominal cavity, cervix, ovaries, and cesarean section scar site.^1^

The classic triad of abdominal pain, amenorrhea, and vaginal bleeding is useful in the diagnosis of ectopic pregnancy. However, only approximately 50% of cases present with all three symptoms, and in the present case, neither amenorrhea nor vaginal hemorrhage was observed.^2^ Ovarian hemorrhage is a condition that needs to be distinguished from ectopic pregnancy. Its underlying mechanisms primarily involve intraovarian hemorrhage, which occurs within the corpus luteum, and extraovarian hemorrhage, which occurs when the corpus luteum ruptures and causes hemorrhage into the abdominal cavity. Both types of hemorrhage can occur simultaneously. These situations result in acute abdominal pain, often accompanied by ovarian enlargement and intraperitoneal hemorrhage. Severe intraperitoneal hemorrhage can potentially lead to shock. Thus, these conditions are very similar to miscarriages or rupture associated with in ectopic pregnancy, making it difficult to differentiate ovarian hemorrhage and ectopic pregnancy by symptoms alone. However, since the presence of chorionic villi leads to the secretion of hCG in ectopic pregnancy, ruling out pregnancy-related disorders, especially ectopic pregnancy, is extremely important for diagnosing and determining treatment strategies. It particularly applies when women of reproductive age present to the emergency department with an acute abdomen. On the other hand, Lee et al. reported an ectopic pregnancy in a patient presenting to the emergency department with an acute abdomen, in whom both urine and serum hCG tests were negative; however, ultrasound showed a large amount of fluid in the abdominal cavity. CT scans showed active hemorrhage from the adnexa and massive intraperitoneal hemorrhage^3^ (Table 1). Similarly, in the present case, the patient was preoperatively diagnosed with ovarian hemorrhage based on a negative qualitative urine hCG test; therefore, laparoscopic surgery was performed. However, intraoperative findings showed no ovarian hemorrhage, and a clinical diagnosis of ectopic pregnancy was made based on the macroscopic findings of fallopian tube enlargement, leading to a change in the surgical procedure. Currently, qualitative urine hCG testing is widely used as an initial screening test due to its simplicity and rapidity. It has a high sensitivity of 99% for pregnancy when the serum β-hCG level exceeds 25 mIU/mL. Therefore, in many clinical settings, a negative qualitative urine hCG test is used to rule out pregnancy in order to determine the feasibility of X-ray examination and differentiate ectopic pregnancy from other conditions (e.g., ovarian hemorrhage and ovarian torsion).^4^ To further improve diagnostic accuracy, it is desirable to perform serum hCG testing in addition to qualitative urine hCG testing. However, many medical institutions, such as ours, make it difficult to obtain serum hCG test results promptly; thus, improvements to the testing system are desired.

hCG is a heterodimeric glycoprotein hormone composed of noncovalently linked α- and β-subunits. It is secreted by placental trophoblast cells. Although its α-subunit is identical to the α-subunit of the three hormones (LH, FSH, and TSH) synthesized by the anterior pituitary gland, the β-subunits are different from one another, allowing for the specific diagnosis of pregnancy by measuring β-hCG. However, although the test reagent used at our hospital is supposed to measure only intact hCG (α- and β-subunits), according to previous reports, the actual target of measurement may vary.

In addition, serum hCG levels in normal pregnancies show a characteristic increase early after fertilization, becoming detectable in urine and serum within 16 days after the LH surge and doubling approximately every 48 to 72 hours thereafter. Meanwhile, the rate of increase in serum hCG levels tends to be lower in ectopic pregnancies than in normal pregnancies. In fact, Mohamad et al. measured the rate of increase in serum hCG levels 48 hours after fertilization. They found that it was 75% in women with ectopic pregnancy compared with 124% in those with normal pregnancy, indicating that only approximately 15% of ectopic pregnancies have serum hCG levels as elevated as normal intrauterine pregnancies.^5^ Therefore, in cases that result in an ectopic pregnancy, the delayed positivity in urine hCG tests may result in a negative qualitative urine hCG test at diagnosis. The mechanisms underlying low hCG levels in ectopic pregnancy include the following: first, the cessation or significant reduction of hCG production itself due to the degeneration or inactivation of trophoblast cells; second, abnormalities in the hCG synthesis process that inhibit the production of normal hCG molecules; third, the reduction in the absolute number of hCG-producing cells due to a quantitative insufficiency of chorionic villi at the site of ectopic implantation; fourth, the production of unusual hCG variants that yield hCG forms that are difficult to detect using standard assays.^6^ Therefore, due to these complex factors, serum hCG levels are often remarkably lower in ectopic pregnancies than in normal pregnancies, and this biochemical feature is an important determinant in clinical diagnosis. Nevertheless, it has also been noted that this contributes to diagnostic complexity.^6^ In fact, as shown in Table 1 summarizing the serum hCG levels, symptoms, and preoperative diagnoses of cases diagnosed with ectopic pregnancy despite a negative qualitative urine hCG test, all cases exhibited low serum hCG levels.^7,8^ In contrast, in the present case, the serum hCG level was relatively high at 314 mIU/mL, and there have been no reported cases of ectopic pregnancy with a negative qualitative urine hCG test showing such high serum hCG levels, suggesting this case’s uniqueness.

In the present case, the patient was rushed to our hospital due to an acute abdomen. Based on peritoneal irritation signs, transvaginal ultrasound findings of a hematoma in the Douglas pouch and a negative qualitative urine hCG test, pregnancy was ruled out. Furthermore, contrast-enhanced CT scans revealed findings suggestive of contrast extravasation around the left adnexa, leading to a preoperative diagnosis of left ovarian hemorrhage. The incidence of ectopic pregnancy with a negative qualitative urine hCG test, as in the present case, is very rare at 1.6%. Factors that contribute to negative qualitative urine hCG tests include menstrual irregularities, ectopic pregnancy, trophoblastic disease, missed abortion, dilution of urine, polyuria, and differences in reagent sensitivity.^9^ In the present case, the fluid challenge administered after the patient was rushed to our hospital may have led to iatrogenic dilution of the urine. Since the urine specimen collected after the fluid challenge showed a significantly low urine specific gravity of 1.004 or less, this urine dilution may have contributed to the false-negative qualitative urine hCG test.^10^ Therefore, in the context of emergency care in the emergency room or infusion therapy to maintain the patient’s general condition, it is necessary to consider the risk of false-negative qualitative urine hCG tests.

## Conclusions

We encountered a patient whose qualitative urine hCG test was resulted as negative, and emergency surgery was initiated based on the diagnosis of ovarian hemorrhage. Ultimately,the case was ultimately handled as an ectopic pregnancy with a change in surgical procedure. The patient was in critical condition due to pain from an acute abdomen, intraperitoneal hemorrhage, and associated coagulopathy, and a delay in surgery could have had a significant impact on the patient’s prognosis. In women of reproductive age presenting with pelvic masses and intraperitoneal hemorrhage, despite a negative qualitative urine hCG test, it is important to always consider ectopic pregnancy as a differential diagnosis. It is critical to make a comprehensive judgment based not only on test results but also on clinical symptoms and imaging findings.

## Disclaimer

Author Haruki Nishizawa is a member of the Editorial Board of Fujita Medical Journal. This author was not involved in the peer-review or decision-making process for this paper.

## Figures and Tables

**Table 1:** Past cases of ectopic pregnancy with negative urine pregnancy test

Author	Age	Clinical Findings	Serum hCG level	Preoperative Diagnosis
Daniilidis, et al. (2014)^7^	36	lower abdominal pain, amenorrhea, syncope tachycardia	13m IU/L	right tubal pregnancy
Sheele, et al. (2016)^2^	35	lower abdominal pain, dyspareunia, dysuria nausea	10 mIU/mL	hemoperitoneum
Hughes, et al. (2017)^8^	25	right lower abdominal pain	15 mIU/mL	ruptured ectopic pregnancy
Paritakul, et al. (2017)^5^	41	lower abdominal pain, amenorrhea, syncope dizziness, hypotension, tachycardia	4.2 mIU/mL	hemoperitoneum
Lee, et al. (2020)^6^	32	lower abdominal pain, syncope, hypotension tachycardia	7.4 mIU/mL	hemorrhagic cyst
Mohamad, et al. (2021)^4^	33	left lower abdominal pain, vaginal bleeding vomiting, tachycardia	not described	not described
Kopelman, et al. (2021)^1^	23	left lower abdominal pain, vaginal bleeding tachycardia	<5 mIU/mL	ruptured right ovarian mass
Dnnphy, et al. (2022)^3^	30s	left lower abdominal pain, vaginal bleeding hypotension, tachycardia	18 IU/L	ruptured ectopic pregnancy

## Supplementary Material

PDF-Japanese
