# Supplementary material for: A case of ectopic pregnancy with a negative urine pregnancy test diagnosed preoperatively as ovarian hemorrhage
Source: Fujita Med J. 2026 May 14;12(3):236–9. doi: 10.20407/fmj.2025-033 (PMC13433101; doi:10.20407/fmj.2025-033)
Supplement: Supplementary file 1 — PDF-Japanese [file fmj-12-236_s1.pdf]

タイトル：卵巣出血と術前診断された尿中妊娠反応陰性の異所性妊娠の 1 例
ランニングタイトル：尿中妊娠反応陰性であった異所性妊娠の 1 例

Ryoma Aoki , MD[1,2], Akira Yasue, MD, PhD[2],Yutaka Torii, MD, PhD[1,2], Ryuichiro Aoki, MD[1], Kazuhiko Tsukada, MD, PhD[2], Haruki Nishizawa, MD, PhD[1]
[1]Department of Obstetrics and Gynecology, Fujita Health University, School of Medicine, Toyoake, Aichi, Japan
[2]Department of Gynecology, Fujita Health University Okazaki Medical Center, Okazaki, Aichi, Japan

Corresponding author: Akira Yasue, MD
Department of Gynecology, Fujita Health University Okazaki Medical Center, 1, Gotanda, Harisaki-cho, Okazaki, Aichi 444-0827, Japan
Tel: 0564-64-8800
E-mail: akira.yasue@sf.commufa.jp

抄録
緒言：異所性妊娠は産婦人科救急疾患の代表的疾患であり、診断には血清・尿中 hCG 測定が重要とされるが、尿中 hCG 定性検査が偽陰性を示すことがある。今回、尿中 hCG 定性検査陰性のため妊娠を除外し卵巣出血の術前診断で手術を施行したが、異所性妊娠と判明した 1 例を経験したので報告する。
症例：19 歳、0 妊 0 産。最終月経 28 日後に下腹部痛で近医を受診し、腹膜刺激症状と腹部 CT 検査で腹水貯留を認めたため当院へ救急搬送された。臍周囲に強い圧痛・反跳痛を認め、経腟超音波検査でダグラス窩に 83mm 大の血腫像を認めた。尿中 hCG 定性検査は陰性であり、造影 CT 検査で左付属器周囲に造影剤の漏出所見を認めたため、左卵巣出血と診断して緊急腹腔鏡手術施行した。しかしながら、術中に左卵管腫大を認めたことから異所性妊娠を疑い、術式を左卵管摘出術へ変更した。術前の血清 hCG 値は 314mIU/ml と上昇しており、病理検査で絨毛組織を確認し左卵管妊娠と診断した。
結語：尿中妊娠反応陰性の異所性妊娠例を経験した。生殖年齢女性の急性腹症では尿中 hCG 定性検査陰性でも異所性妊娠の可能性を常に考慮し、慎重なインフォームド・コンセントが必要である。

キーワード：尿中妊娠反応陰性、異所性妊娠、卵巣出血

【緒言】

異所性妊娠は、産婦人科領域における代表的な産科救急疾患である。その発症頻度は全妊娠の約 1-2%とされ、妊娠可能年齢の女性における急性腹症の重要な鑑別診断の一つとして位置づけられている。異所性妊娠の発生部位は卵管が約 ９６％を占め、その他卵巣、腹腔、頸管などとされている[1]。異所性妊娠の臨床症状は、典型的には無月経、下腹部痛、性器出血の三主徴を呈するが、これらすべてが揃うことは約 50%程度とされ、病態は非常に多彩で診断の苦慮する場合があり、さらに卵管流産や卵管破裂をきたした場合には、急性腹症として発症し、迅速な診断と適切な治療が求められる点が問題である。

一方、異所性妊娠と鑑別を要する疾患として卵巣出血が挙げられ、本症は排卵期や黄体期さらには妊娠中も含めて卵巣内または卵巣外に出血する疾患であり、異所性妊娠と同様に腹腔内出血から急性の下腹部痛と貧血をきたす。

異所性妊娠と卵巣出血の鑑別診断の際には、妊娠の存在を確認するために血清または尿中ヒト絨毛性ゴナドトロピン（hCG）の測定が重要で、特に血清 hCG は高い感度と特異度を有している。しかしながら、尿中 hCG 定性検査はまれに偽陰性を示すことがあり、血清 hCG と比較して感度が劣るため、血清 hCG が陽性であっても尿中 hCG 定性検査が陰性となる症例も報告されており [2]、こうした場合には妊娠の可能性が除外されやすく、両者の鑑別が困難となることがある[3]。

今回われわれは、術前の尿中 hCG 定性検査が陰性であったため妊娠の可能性が除外され、卵巣出血の診断で緊急手術を施行したところ、術中所見により異所性妊娠と判明した症例を経験した。本症例は、hCG 陰性例における異所性妊娠の診断の困難さと、詳細な病歴聴取および術中所見の重要性を示唆する教訓的な症例であり、文献的考察を加えて報告する。

【症例】

19 歳、0 妊 0 産。既往歴・家族歴に特記すべき事項なし。月経周期は 28 日型で整。最終月経から 28 日目に下腹部痛を訴えて近医婦人科を受診したが、特に異常を指摘されなかった。しかし同日夜間に再度腹痛が出現したため、翌日に近医消化器内科を受診したところ、腹膜刺激症状と腹部 CT 検査で腹水貯留所見を認めたとのことで、当院へ救急搬送となった。

受診時の意識は清明で、血圧 109/60mmHg、脈拍数 111 回/分であった。身体所見では、腹部の臍周囲に強い圧痛と反跳痛を認めた。経腟超音波検査では、子宮内膜の肥厚と胎嚢像を認めず、ダグラス窩には 83mm 大の血腫様腫瘤像が描出された。血液検査では、白血球数 17,300/μL、CRP 1.11mg/dL、Hb 10.2g/dL と軽度の炎症と貧血所見を呈し、凝固系検査では PT-INR 1.18 と軽度延長していた。妊娠の可能性を除外するため尿中 hCG 定性検査で陰性を確認した後に造影 CT 検査を施行したところ、上腹部に至る腹水の貯留を認め、左付属器周囲に造影剤の血管外漏出を疑う所見を認めたが、右卵巣は正常所見であった（図 1)。尿中 hCG 定性検査陰性であったため、この時点では異所性妊娠の可能性を除外し、左卵巣出血と診断して緊急腹腔鏡手術を施行した。術中所見では、ダグラス窩に血腫の貯留を認め、腹腔内出血量は約 500g であった（図２)。両側卵巣に腫大や出血を認めず、卵巣出血は否定的であったが、左卵管の腫大を認め、その肉眼所見が異所性妊娠に特徴的な卵管腫大の所見と類似していたことから、尿中 hCG 定性検査が陰性であったものの異所性妊娠の可能性を考慮した。術中に患者家族へ診断と術式の変更についてインフォームドコンセントを行い、腹腔鏡下左卵管摘出術へ術式を変更した。摘出標本では肉眼的に明らかな絨毛成分は認めなかった。術後、術前検体での血清 hCG 値を確認したところ 314mIU/mL であり、病理組織学的検査でも絨毛組織を確認し、最終的に左卵管妊娠の診断に至った（図 3)。患者は術後経過良好で退院となった。

術後 1 か月後の外来受診時に血清 hCG 値が測定感度以下であることを確認し、治療を終了した。

【考察】

異所性妊娠とは受精卵が子宮腔外に着床する妊娠で、腹腔内出血を引き起こし、ショックから死に至る可能性のある疾患である。近年、妊産婦の高齢化をはじめ既往帝王切開や生殖補助医療による妊娠の増加により、異所性妊娠の発生率は約２％に増加している。発生部位は卵管が最も多く、全異所性妊娠の約 96%を占め、その他、腹腔内、子宮頸部、卵巣、帝王切開瘢痕部などが挙げられる[1]。

異所性妊娠の診断には、古典的な三徴候は腹痛、無月経、性器出血が有用であるが、これらの症状が揃うのは約 50%の症例に過ぎないとされ、本症例でも無月経や性器出血は認めなかった[2]。また、異所性妊娠と鑑別を要する疾患としては卵巣出血が挙げられ、その病態には主に黄体内に出血する卵巣内出血と、これが破裂して腹腔内に出血をきたす卵巣外出血、さらには両者が共存する場合があり、いずれも卵巣の緊満や腹腔内出血に伴う急性腹症をきたし、腹腔内出血量が多い場合はショックに至る。したがってこれらの病態は異所性妊娠の流産や破裂と非常に類似しており、症状のみで両者を診断することは困難である。ただし、異所性妊娠では、絨毛の存在によって hCG が分泌されることから、少なくとも生殖可能年齢の女性が急性腹症で救急外来を受診した場合、妊娠関連疾患、特に異所性妊娠の除外は診断・治療方針決定において極めて重要である。その一方で、Lee らは、急性腹症で救急外来を受診した症例において、尿および血清 hCG 検査がともに陰性であったにも関わらず、超音波検査で腹腔内への大量の液体貯留を認め、CT 検査では付属器からの活動性出血と大量の腹腔内出血が確認された異所性妊娠の存在を報告している [3]。（表１）本症例でも同様に術前診断では尿中 hCG 定性検査が陰性であったことから、卵巣出血の診断の下で腹腔鏡手術が実施されたが、卵巣出血はなく、肉眼的な卵管の腫大所見から異所性妊娠との臨床診断の下で術式を変更することとなった。現在、尿中 hCG 定性検査は簡便性と迅速性から初期スクリーニング検査として広く用いられており、血清 β-hCG 値が 25 mIU/mL を超える場合に妊娠の感度が 99%と高いことから、多くの臨床現場で尿中 hCG 定性検査陰性の結果をもって妊娠を除外することによって、X 線検査の可否や異所性妊娠と鑑別を要する疾患（卵巣出血、卵巣茎捻転など）との鑑別に用いられている[4]。ただし、診断精度をさらに向上させるためには、尿中 hCG 定性検査に加えて血清 hCG 検査を併行することが望ましいが、当施設のように血清 hCG 検査の迅速な結果取得が困難な医療機関も多く、検査体制の整備が求められる。

hCG は、非共有的に結合した α および β-サブユニットからなるヘテロ二量体糖蛋白質ホルモンであり、胎盤の栄養膜細胞から分泌される。このうち、α-サブユニットは下垂体前葉によって合成される 3 つのホルモン(LH, FSH, TSH)の α-サブユニットと同一であるが、これらの β-サブユニットはそれぞれ異なっており、β-hCG の測定により妊娠の特異的診断が可能となる。ところが、当施設で採用している検査試薬では intact hCG（α および β-サブユニット)のみ測定していることになっているが、既報によると実際の測定対象は不明である。また、正常妊娠における血清 hCG 値は、受精後早期に特徴的な上昇を示し、LH サージ後

16 日以内に尿と血清中で検出可能となり、その後、約 48～72 時間ごとに倍増する。一方で、異所性妊娠の場合には、血清hCG値の増加率は正常妊娠よりも低い傾向があるとされている。実際に、Mohamad らは、受精後 48 時間後の血清 hCG 値の増加率を測定したところ、異所性妊娠の女性では 75%であったのに対し、正常妊娠では 124%であり、異所性妊娠の血清 hCG 値が正常子宮内妊娠と同様に上昇するのは約 15%に過ぎないことを示している[5]。したがって、異所性妊娠に至る症例では、尿中 hCG の陽性化が遅延することにより、診断時の尿中hCG定性検査が陰性と判断されてしまう可能性がある。このような、異所性妊娠がhCG低値となるメカニズムとして、第一に、栄養膜細胞の変性や不活化により、hCG の産生そのものが停止または著しく減少する、第二に、hCG の合成過程における異常により、正常なhCG分子の産生が阻害される、第三に、異所性着床部位における絨毛組織の量的不足により、hCG 産生細胞の絶対数が減少する、第四に、通常とは異なるhCG バリアントの産生により、標準的な測定法では検出されにくい形態の hCG が産生される可能性などが考えられている[6]。したがって、これらの複合的な要因により、異所性妊娠では血清hCG値が正常妊娠と比較して著明に低値を示すことが多く、この生化学的特徴が臨床診断における重要な判断材料となるが、逆にこれが診断の複雑性を増す要因となると指摘されている[6]。実際に、尿中 hCG 定性検査が陰性であったにもかかわらず異所性妊娠と診断された症例の血清 hCG 値、症状、術前診断をまとめて示すが（表１） いずれも血清 hCG 値が低値を示していた[7, 8]。ところが、今回の症例では血清 hCG 値が 314 mIU/ml と比較的高値を呈しており、こうした血清hCG値を示す尿中hCG定性検査陰性の異所性妊娠症例の報告はなく、これが本症例の特異性を示唆している。

本症例では急性腹症により救急搬送され、腹膜刺激症状と経腟超音波検査によるダグラス窩に血腫像を認め、尿中hCG定性検査が陰性であったことから妊娠は否定的と判断し、さらに造影 CT 検査で左付属器周囲に血管外漏出を疑う所見を認めたため、左卵巣出血と術前診断した。ところが、本症例のように尿中hCG定性検査陰性の異所性妊娠の発生率は 1.6%と非常に稀であり、尿中 hCG 定性検査陰性となる要因として、月経不順、異所性妊娠、絨毛性疾患、稽留流産、希釈尿、多尿、試薬の感度差などが挙げられている[9]。今回の症例では、救急搬送後に施行された輸液負荷により医原性の希釈尿が生じた可能性があり、実際に輸液負荷後に採取した尿検体においても尿比重が 1.004 以下と著明な低値を示していたことから、この希釈尿が尿中hCG定性検査における偽陰性の一因となった可能性がある[10]。したがって、救急外来での救命救急や全身状態の維持を目的とした輸液療法が実施されている状況下では、尿中hCG定性検査が偽陰性となるリスクを念頭に対応する必要があると考えられた。

今回、尿中hCG定性検査が陰性と判断され、卵巣出血の診断の下で緊急手術を開始したが、最終的には異所性妊娠として術式を変更して対応した症例を経験した。患者は、急性腹症による疼痛と、腹腔内出血ならびにそれに伴う凝固異常によって重篤な状態であり、手術時期の遅延は患者の予後に重大な影響を与える可能性があったが、生殖年齢女性にお

ける骨盤内腫瘤や腹腔内出血の症例では、尿中 hCG 定性検査が陰性であっても異所性妊娠を鑑別診断として常に念頭に置くとともに、検査結果に加えて臨床症状や画像所見を総合的に判断する姿勢が求められる。

引用文献

１．Kopelman ZA, Keyser EA, Morales KJ. Ectopic pregnancy until proven otherwise… even with a negative serum hCG test: A case report. Case Reports in Women's Health 2021；30: e00288.

２．Sheele JM, Bernstein R, Counselman FL. A Ruptured Ectopic Pregnancy Presenting with a Negative Urine Pregnancy Test. Case Rep Emerg Med　2016；2016: 7154713.

３．Dunphy L, Arias Rey C, Arshad I, Hapangama DK. Ruptured chronic ectopic pregnancy presenting with a negative urine pregnancy test. BMJ Case Rep　2022；15: e245742.

４．Mohamad F, Yahya AS, Abdul Rashid A, Devaraj NK, Abdul Manap AH. A life-threatening condition—ruptured ectopic pregnancy with negative urine pregnancy test: A case report. Malays Fam Physician　2021；16: 121-123.

５．Paritakul P. Ruptured Tubal Pregnancy with a Negative Urine Pregnancy Test and Serum Beta Human Chorionic Gonadotropin. Thai Journal of Obstetrics and Gynaecology　2017；25: 130-135.

６．Lee TH, Chou CC, Chang CF, Lin YR, Chen WL. A Rare Case of Ruptured Ectopic Pregnant Woman Present With Double Negative Pregnant Test. Journal of Acute Medicine　2020；10: 126-128.

７．Daniilidis A, Pantelis A, Makris V, Balaouras D, Vrachnis N. A unique case of ruptured ectopic pregnancy in a patient with negative pregnancy test - a case report and brief review of the literature. HIPPOKRATIA　2014；18: 282-284.

８．Hughes M, Lupo A, Browning A. Ruptured ectopic pregnancy with a negative urine pregnancy test. Proc (Bayl Univ Med Cent)　2017；30: 97-98.

９．Hatase T, et al. Problems with low-unit pregnancy diagnostic aids. New Drugs and Clinical Practice 2003; 134-42.

１０．　Kleinschmidt S, Dugas JN, Nelson KP, Feldman JA. False negative point-of-care urine pregnancy tests in an urban academic emergency department: a retrospective cohort study. JACEP Open　2021；2: e12427.

Table 1 ：尿中妊娠反応陰性の異所性妊娠の過去の症例

Author	Age	Clinical Findings	Serum hCG level	Preoperative Diagnosis
Daniilidis,et al(2014)[7]	36	lower abdominal pain, amenorrhea, syncope tachycardia	13m IU/L	right tubal pregnancy
Sheele,et al(2016)[2]	35	lower abdominal pain, dyspareunia, dysuria nausea	10 mIU/mL	hemoperitoneum
Hughes,et al(2017)[8]	25	right lower abdominal pain	15 mIU/mL	ruptured ectopic pregnancy
Paritakul,et al(2017)[5]	41	lower abdominal pain, amenorrhea, syncope dizziness, hypotension, tachycardia	4.2 mIU/mL	hemoperitoneum
Lee,et al(2020)[6]	32	lower abdominal pain, syncope, hypotension tachycardia	7.4 mIU/mL	hemorrhagic cyst
Mohamad,et al(2021)[4]	33	left lower abdominal pain, vaginal bleeding vomiting, tachycardia	not described	not described
Kopelman,et al(2021)[1]	23	left lower abdominal pain, vaginal bleeding tachycardia	<5 mIU/mL	ruptured right ovarian mass
Dnnphy,et al(2022)[3]	30 s	left lower abdominal pain, vaginal bleeding hypotension, tachycardia	18 IU/L	ruptured ectopic pregnancy

Figure 1a

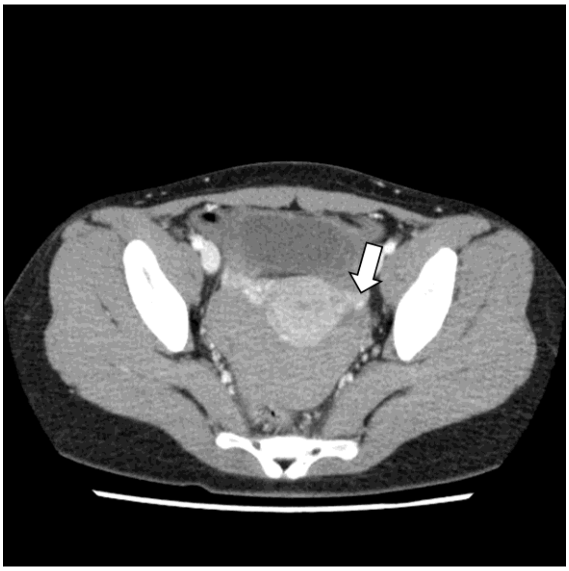

Figure 1b

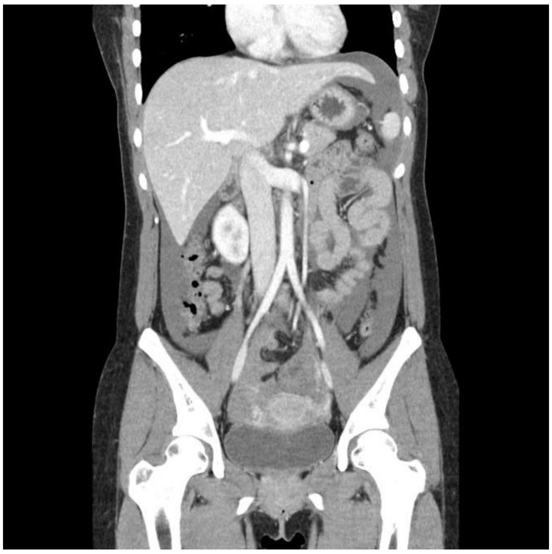

Figure 2

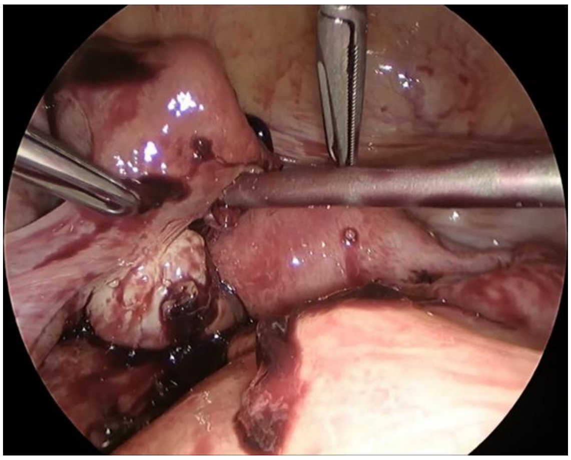

Figure 3

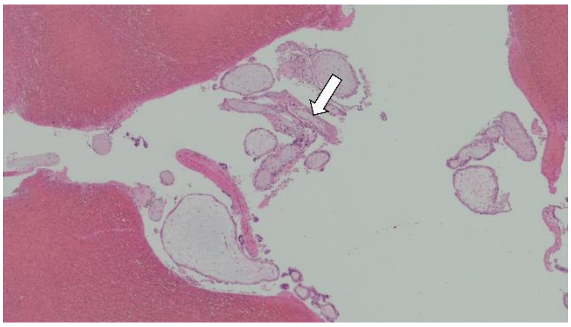

Figure1　腹部造影 CT 検査：

a) 左付属器周囲に造影剤の血管外漏出（⇨）を疑う所見を認めた。

b) 肝・脾周囲まで腹水の貯留を認めた。

Figure 2　 手術所見

開始時、ダグラス窩に多量の血腫貯留を認めた。

左卵管所見：左卵管腫大を認めた。

Figure 3　 病理組織所見

腫大した左卵管内に少量の絨毛組織（⇨）を認めたが毛細血管の発達や胎児成分は確認

されなかった。
